# Lessons from a “Failed” Experiment: Zinc Silicates with Complex Morphology by Reaction of Zinc Acetate, the Ionic Liquid Precursor (ILP) Tetrabutylammonium Hydroxide (TBAH), and Glass

**DOI:** 10.3390/ma1010003

**Published:** 2008-08-29

**Authors:** Zhonghao Li, Yaroslav Z. Khimyak, Andreas Taubert

**Affiliations:** 1 Institute of Chemistry, University of Potsdam, Karl-Liebknecht-Str. 24-25, Building 26, D-14476 Golm, Germany; 2 Department of Chemistry, University of Liverpool, Liverpool L69 7ZD, UK; E-mail: khimyak@liv.ac.uk; 3 Max-Planck-Institute of Colloids and Interfaces, D-14476 Golm, Germany

**Keywords:** Ionic liquids, willemite, hemimorphite, catalysis, high surface area, inorganic sponges

## Abstract

At elevated temperatures, the ionic liquid precursor (ILP) tetrabutylammonium hydroxide reacts with zinc acetate and the glass wall of the reaction vessel. While the reaction of OH^-^ with the glass wall is not surprising as such and could be considered a failed experiment, the resulting materials are interesting for a variety of applications. If done on purpose and under controlled conditions, the reaction with the glass wall results in uniform, well-defined hemimorphite Zn_4_Si_2_O_7_(OH)_2_·nH_2_O and willemite Zn_2_SiO_4_ microcrystals and films. Their morphology can be adjusted by variation of the reaction time and reaction temperature. The hemimorphite can be transformed to Zn_2_SiO_4_ via calcination. The process is therefore a viable approach for the fabrication of porous films on glass surfaces with potential applications as catalyst support, among others.

## 1. Introduction

Because of their interesting structures, properties, and applications, biological and synthetic nanoscale materials are one of the top research areas these days [1,2,3,4,5,6,7,8,9,10,11,12,13]. Nanoparticles in particular have gained tremendous attention. Typically, aqueous and organic solvents have been used for inorganic nanoparticle synthesis in the laboratory [14,15]. Ionic liquids (ILs) have in the recent past also gained increasing attention [16,17,18,19]. ILs have successfully been used for the synthesis of inorganic materials and surfaces as well [16,17,19,20,21,22]. In some cases, ILs lead to materials that are not accessible via other methods [20,22,23,24,25,26,27,28] and in a few cases, ILs have even been shown to be solvents for metal oxides [29]. For example, the reaction of titanium alkoxides with traces of water in ILs provide easy access to hollow TiO_2_ spheres [25] and ionothermal synthesis yields well-defined metal phosphates [22,26,30,31,32,33]. ILs also enable the controlled, low temperature synthesis of anatase and rutile [27], ceria and silica with well-defined pores [16,34,35], and different alumina phases [36]. ILs are therefore an interesting reaction medium for the fabrication of inorganic nanostructures. As a result, inorganic synthesis in ILs, including mixtures of ILs with other solvents like water or toluene, has been studied intensely over the last years [16,17,19,22,30,37].

More recently, ILs and ionic liquid crystals (ILCs) [38] have been designed such that they act as solvent-reactant-templates, that is, the IL is the solvent, the template, and the reactant for the controlled formation of an inorganic material at the same time. These reactive IL(C)s have been termed ionic liquid crystal precursors (ILCPs) [39] and ionic liquid precursors (ILPs) [40], respectively. For example, we have used ILCPs for the fabrication of CuCl platelets [39,41,42]. We have also shown that crystalline ILCP analogs are precursors for Au platelets and that tetrabutylammonium hydroxide (TBAH) is an ILP for various metal oxides [43,44,45,46,47]. Other research groups have modified our original approach [39] to fabricate inorganics with well-defined properties from ILPs and ILCPs [40,48,49,50].

Of particular interest to the current work is a report by Zhu *et al*., who have shown that alkylammonium zinc complex/tetramethylammonium hydroxide mixtures are ILPs for the ionothermal synthesis of well-defined ZnO microparticles [40]. We have extended this approach and shown that the ILP TBAH, that is, the butyl analog of the hydroxide compound used by Zhu *et al*., can be used for the fabrication of ZnO/carbohydrate hybrid materials [46]. Furthermore, unique hollow zinc oxide mesocrystals can also be grown by reaction of TBAH with zinc acetate [44].

The current paper shows that the ILP TBAH also leads to zinc silicates with uniform and complex morphologies, albeit (up to now) not to single-phase materials. The zinc silicates form if the temperature of the reaction medium (the ILP), the vapor phase above the IL, and especially the glass wall of the flask where the reaction is conducted, are significantly higher than in a previous study, where pure ZnO with unique hollow rod morphologies was obtained [44]. The current approach could for example be useful for the in situ modification of glass tubes or surfaces with a catalytically active material, hemimorphite or willemite.

## 2. Results and Discussion

In short, TBAH (5 g) was heated in a 25 mL flask to ca. 50 °C until the IL was liquid. Upon cooling to ca. 30 °C, Zn(OAc)_2_ dihydrate (50 mg) powder was added. The flask was immersed in the oil bath such that also the volume of the flask above the reacting liquid was immersed in the oil bath. After twelve hours, a thick (up to ca. 0.5 mm) white film formed on the glass walls of the flask. After 30 hours, the white solid was recovered via centrifugation, washing with water and ethanol, and drying.

**Figure 1:**
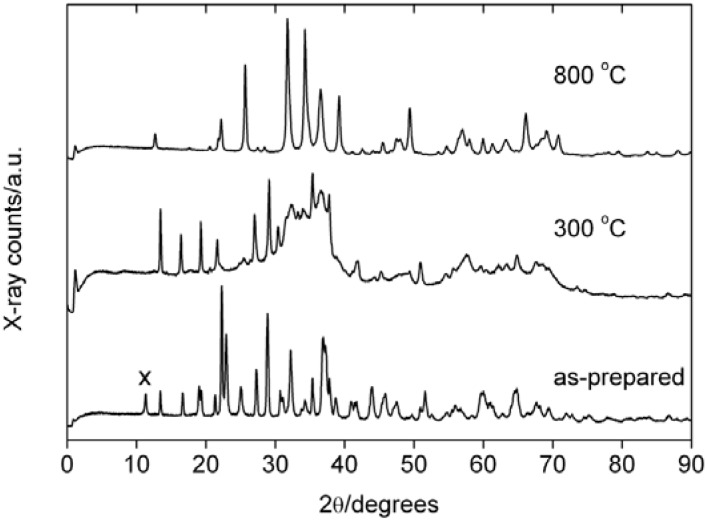
XRD patterns of a precipitate obtained after 30 hours of reaction time and the same sample after calcination at 300 and 800 °C, respectively. (x) denotes the γ-Zn(OH)_2_ 110 reflection.

Figure 1 shows representative X-ray diffraction (XRD) patterns of several samples. XRD shows that after 30 hours of reaction at 130 to 135 °C (temperature of the glass wall of the reaction vessel), the precipitate appears well-crystallized, which is evidenced by the narrow reflections. Analysis of the powder pattern however reveals that the sample is a mixture of hemimorphite Zn_4_Si_2_O_7_(OH)_2_·nH_2_O (JCPDS 00-05-0555), willemite (JCPDS 00-037-01485), and γ-Zn(OH)_2_ (JCPDS 00-020-1437). The presence of zinc hydroxide is somewhat surprising, as the reaction temperature is higher than in our previous work [44,46], where single phase zincite (ZnO) was obtained at somewhat lower temperatures.

Calcination between 300 and 500 °C leads to a rather complex mixture of hemimorphite, zincite ZnO, and willemite Zn_2_SiO_4_. Calcination at 600 °C completely transforms the hemimorphite precursor into willemite. Here, the reflections in the XRD patterns are rather broad, which indicates the presence of small crystallites, a large number of defects, or the presence of multiple structures that could not be identified.

The reflection at 11° 2θ in the XRD pattern of the as-precipitated sample is the (110) reflection of γ-zinc hydroxide. Its absence after calcination even at only 300 °C shows that, unlike the silicates, the transformation of zinc hydroxide into zincite can, as expected, be achieved at relatively low temperatures.

Calcination at 700 °C and higher leads to willemite and zincite and the XRD patterns show relatively narrow and well-developed reflections. This indicates that here the crystals have much larger coherence lengths and much less defects than samples calcined at lower temperature. These data also clearly show that unlike the transformation of Zn(OH)_2_ to ZnO, the transformation of the hemimorphite component of the sample to willemite requires higher temperatures.

**Figure 2:**
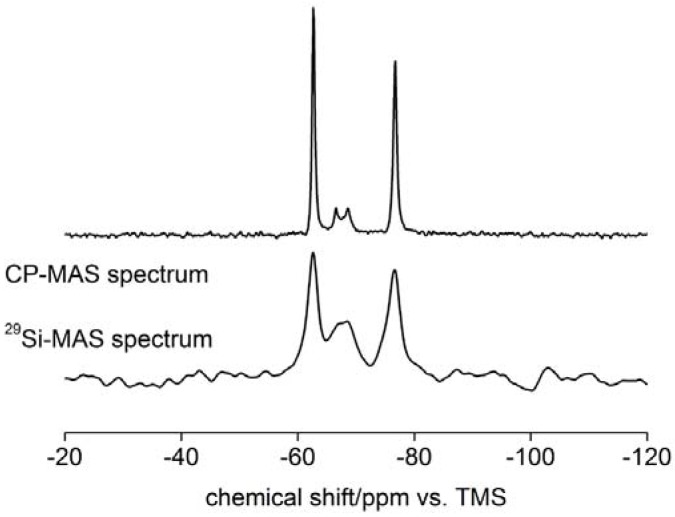
^29^Si-MAS and ^1^H-^29^Si CP MAS SSNMR spectrum of an as-prepared sample.

Figure 2 shows ^1^H-NMR and ^29^Si magic angle spinning (MAS) NMR spectra of an as-prepared sample. While XRD experiments suggest that the as-prepared sample consists of the compounds hemimorphite Zn_4_Si_2_O_7_(OH)_2_·nH_2_O, willemite Zn_2_SiO_4_, and γ-zinc hydroxide, solid state NMR (SSNMR) clearly shows that there are several species present. The peak at ‑76.8 ppm can be assigned to Q^1^ silicate units in hemimorphite [51] and the peak at –66 ppm is due to the presence of willemite [52]. The downfield resonances fall in the chemical shift range characteristic of Q^0^ silicate units, but the assignment of the peak at -62.7 ppm is unclear.

The ^29^Si chemical shifts in zinc silicates depends on the degree of condensation of the silicate units, the Si-O-Si angles and the number of Zn atoms in the first coordination sphere of silicon [53,54,55,56]. The very slow ^1^H-^29^Si CP/MAS kinetics with long T_cp_ times suggests that the peaks at -62.7 and -76.8 ppm correspond to silica units which do not bear protons in close proximity to silicon atoms. In contrast, the peaks at -66.5 and ‑68.5 ppm feature much faster CP-kinetics. This indicates much stronger ^1^H-^29^Si heteronuclear dipolar interactions which can be explained by the shorter ^1^H-^29^Si distances.

The ^1^H source for the as-synthesized materials mainly consists of Si-OH protons and protons from water molecules in the structure as suggested by the values of ^1^H chemical shifts for the main broad resonances. These values are consistent with the previously reported data for silicate-based minerals [57].

**Figure 3:**
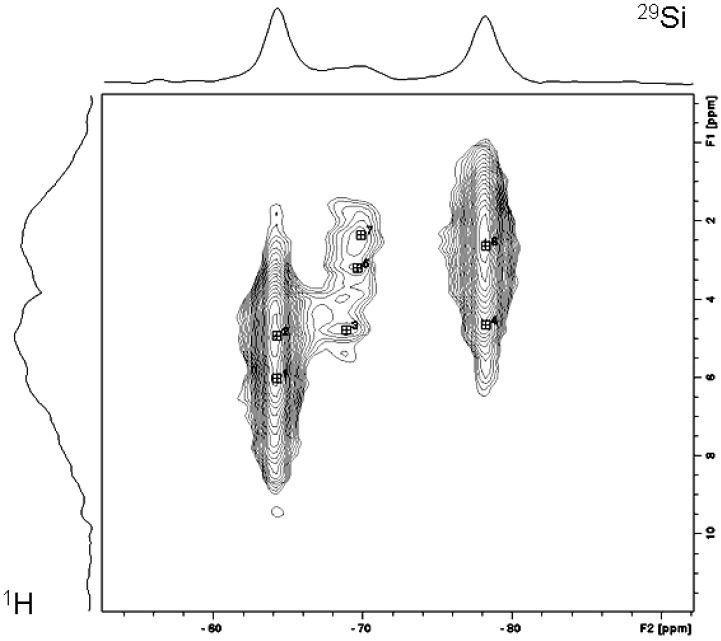
^1^H-^29^Si Heteronuclear Correlation (HETCOR) spectra.

The nature of the connectivities in the precipitate was probed using ^1^H-^29^Si Heteronuclear Correlation (HETCOR) spectroscopy (Figure 3). Two broad lines in the ^1^H dimension correlate with different ^29^Si sites. The main ^29^Si resonances at -62 and -78 ppm show most intense correlating cross-peaks with different proton resonances, indicating that these silicon sites are present in different structures. On the other hand the ^29^Si peaks at -78 and -68 ppm show the most intense cross-peaks with identical ^1^H sites. A similar pattern is observed for the ^29^Si sites at -62 and -64 ppm indicating that these peaks represent identical phases. One should bear in mind that the broadness of ^1^H lines makes the assignment additionally difficult.

**Figure 4:**
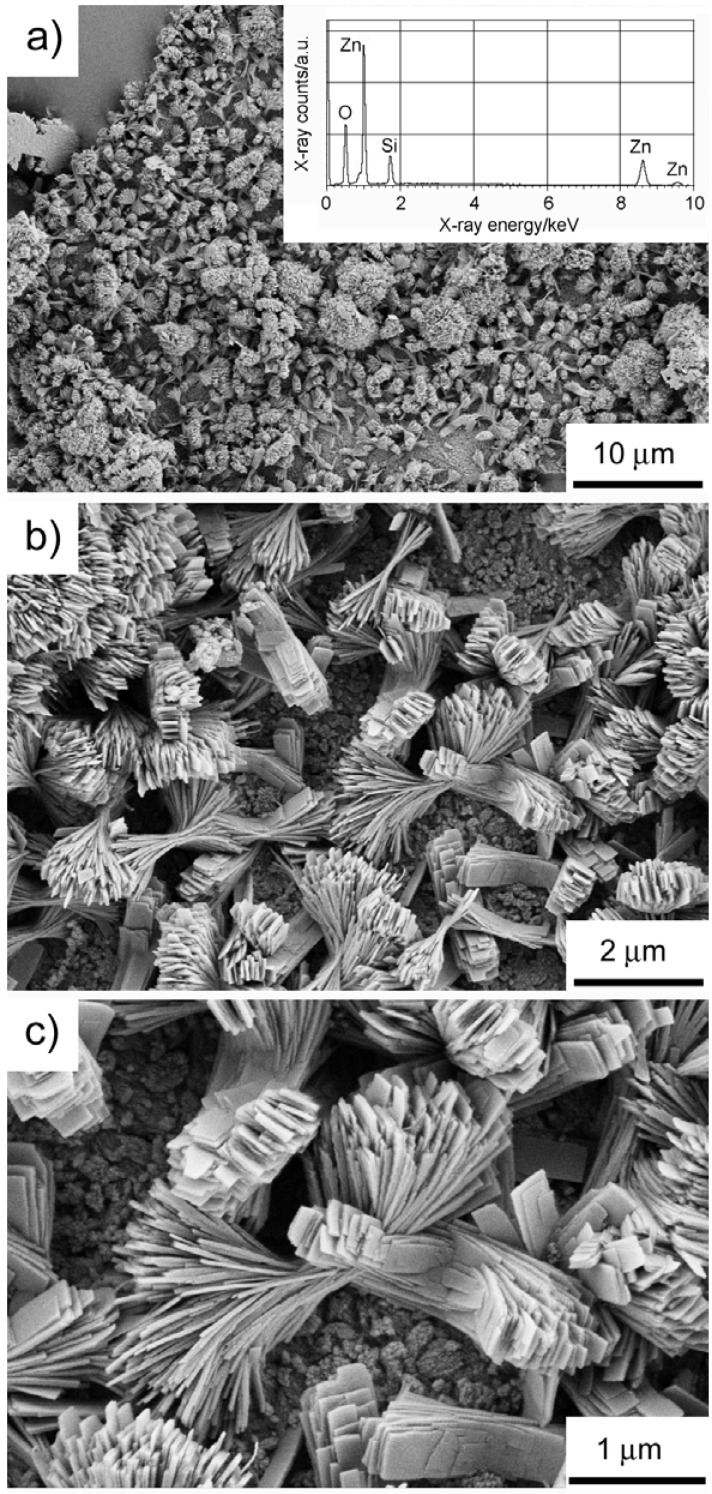
SEM of the as-precipitated zinc silicate after 20 hours of reaction time. Inset: EDX spectrum.

Energy dispersive X-ray spectroscopy (EDXS, see Figure 4) only detects Si, Zn, and O and hence supports our XRD and SSNMR structure assignment. The chemical composition of the precipitates has been further confirmed by atomic absorption spectroscopy (AAS). Table 1 summarizes the sample compositions as determined from SSNMR and XRD experiments.

**Table 1:** Crystal structures as a function of calcination temperature T_calc._ determined from XRD and SSNMR.

T_calc._	Phase 1	Phase 2	Phase 3
---	Zn_4_Si_2_O_7_(OH)_2_*n H_2_O	Zn_2_SiO_4_	γ-Zn(OH)_2_
300	Zn_4_Si_2_O_7_(OH)_2_*n H_2_O	Zn_2_SiO_4_	ZnO
400	Zn_4_Si_2_O_7_(OH)_2_*n H_2_O	Zn_2_SiO_4_	ZnO
500	Zn_4_Si_2_O_7_(OH)_2_*n H_2_O	Zn_2_SiO_4_	ZnO
600		Zn_2_SiO_4_	ZnO
700		Zn_2_SiO_4_	ZnO
800		Zn_2_SiO_4_	ZnO
1000		Zn_2_SiO_4_	ZnO

Figure 4 shows scanning electron microscopy (SEM) images of the precipitates. SEM reveals that the samples have a rather complex morphology after 20 hours of reaction time. The as-prepared precipitate consists of near-rectangular plate-like crystals, which have grown into a dumbbell-like overall morphology. The dumbbells are composed of individual, well-developed, rectangular plates with lengths of up to several hundred nanometers. This morphology is strikingly different from zinc silicates reported in the literature. Roy *et al*. reported the formation of spherical particles with a diameter of ca. 50 nm [58]. Wei *et al*. reported the formation of wires with a length of up to several micrometers [59].

In part this different morphology can be assigned to the different synthesis process. Our materials are grown in a hydrated ionic liquid at moderate temperatures and ambient pressure. The other materials are made via chemical vapor synthesis from an organometallic single-source precursor [58] or via a thermal evaporation and condensation process [59]. This rather different approach makes a comparison difficult, yet it also shows that our approach is a viable option for the fabrication of zinc silicates with completely different morphological characteristics.

The complex shape of the precipitates with plates that grow in the same general direction, but start to branch with increasing distance from the center, suggests that the particles form to some extent via self-similar growth. This is qualitatively similar to fluorapatite, calcium carbonate, barium carbonate, and barium sulfate, which have also been reported to form via self-similar growth in the presence of polymeric additives. For example, a stepwise growth of fluorapatite aggregates in a gelatin matrix from rods to peanuts, dumbbells, notched spheres, and finally spheroids with a complex inner structure has been reported by Kniep and coworkers [60,61,62,63,64,65,66,67,68]. The fractal branching of successive generations of rods, that is, the self-similar growth, and the overall symmetry of the self-assembled aggregates have been explained with intrinsic electric fields, which take over control of aggregate growth [60].

This mechanism was also used to explain the growth of CaCO_3_ and BaCO_3_ in the presence of polymeric additives [69,70,71,72,73,74,75,76,77,78]. In addition, Cölfen *et al*. suggest that the probability of nucleation on the side-surfaces of rodlike or ellipsoidal primary particles plays an important role in determining the final morphology of BaSO4 particles [79]. According to this, the outward bending of a new generation of subcrystals is due to nucleation and growth of the new crystals on the already existing side faces of subcrystals that have formed earlier. Cölfen and colleagues further suggest that too high tilt angles weaken the “templating” by the existing subcrystal, and that this is the limiting factor for the final particle morphology.

Here, SEM provides clear evidence for a self-similar growth and the current paper is therefore the first example of self-similar crystallization from an IL. To date the driving force for the formation of the complex architecture of the precipitates is not clear yet. However, willemite has a polar crystal lattice [80], which could account for a similar growth mode. Hemimorphite also has a polar lattice [81], and (in geological species) tends to grow as thin, tabular crystals that show hemimorphism (different crystal terminations on either end of the prism form). Also in geological samples, hemimorphite often grows as fan or sheaf-like crystals [80]. This is consistent with the morphology of our samples (although on a different length scale), which suggests that the hemimorphite component in the samples controls the morphology of the precipitates observed in the SEM. This is further supported by the more intense hemimorphite signal at -76.8 ppm in the ^29^Si-SSNMR spectra.

**Figure 5:**
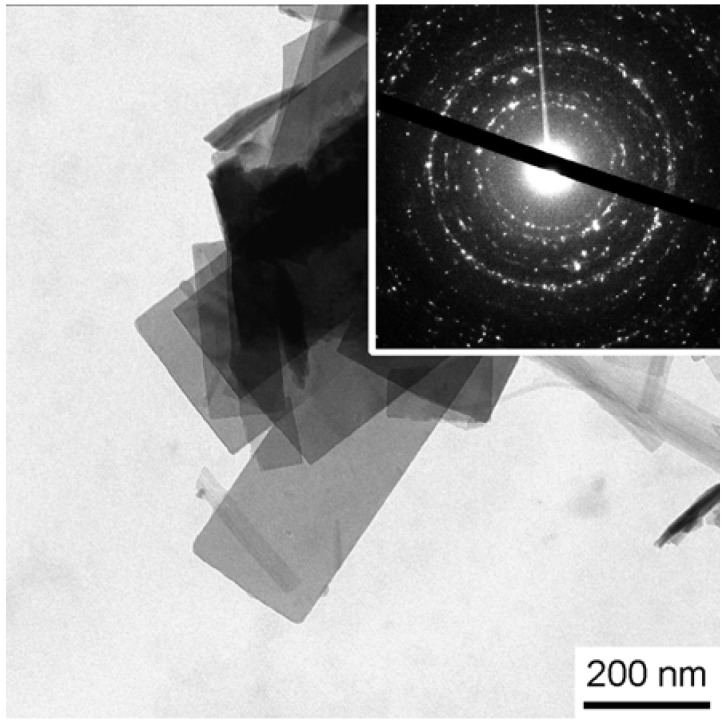
TEM image and electron diffraction pattern of platelets obtained after 30 hours of reaction time.

Figure 5 shows a transmission electron microscopy (TEM) image of a sample isolated after 30 hours. The individual platelets have a well-developed shape with sharp edges. The uniform gray level of individual particles indicates that their thickness is essentially the same over the whole platelet and the absence of bend contours shows that the platelets are, unlike many metal nanoplates, stiff enough to not bend significantly on the TEM grid. Electron diffraction confirms the crystallinity of the sample. The morphology of the plates observed in both SEM and TEM experiments strongly suggests a single crystalline nature of the individual plates.

The presumed single crystal nature of the plates raises the question whether or not individual plates are composed of both silicates and γ-zinc hydroxide or if each plate is a single phase compound. However, attempts to study an individual plate by electron diffraction to determine whether each plate is a single crystal and a single phase compound have so far failed due to the high stability of the aggregated particles and the corresponding difficulty to isolate individual platelets.

**Figure 6:**
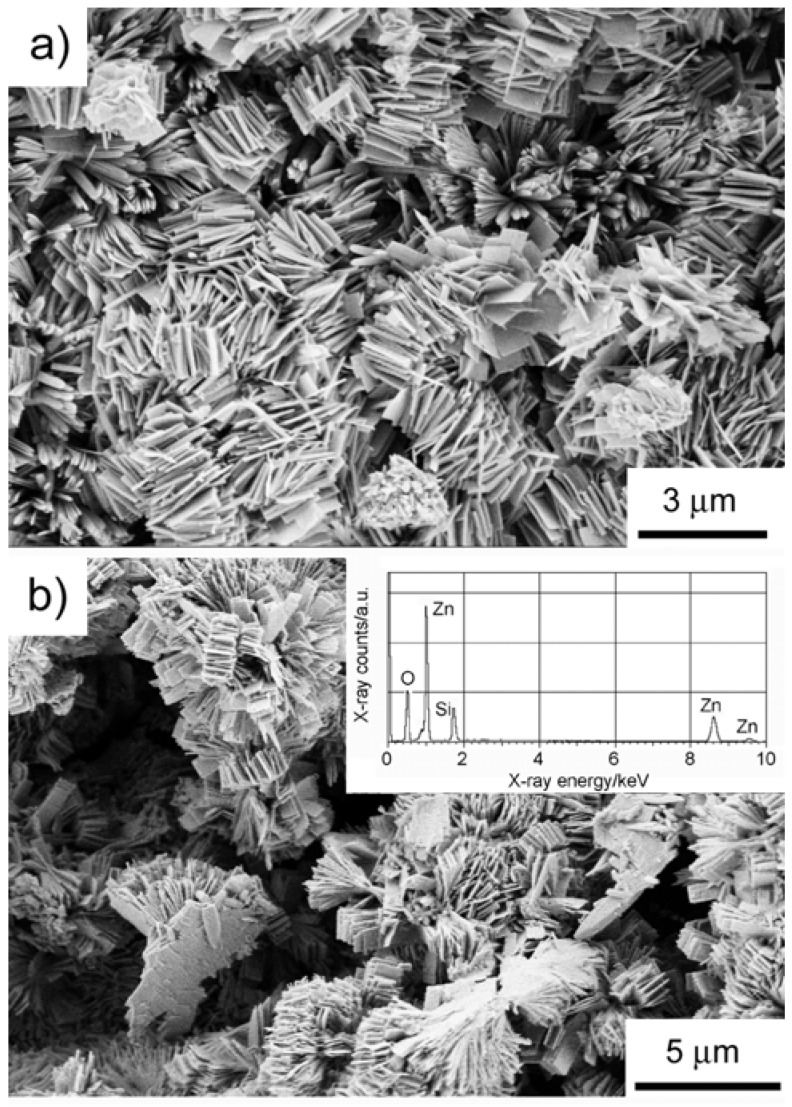
SEM images after calcination at (a) 300 and (b) 800 °C showing that the morphology of the particles does not change upon calcination. Inset: EDX spectrum of the sample calcined at 800 °C.

Figure 6 shows SEM images of calcined samples. SEM shows that the particle morphology is only weakly affected by calcination. The only, rather qualitative, difference is that the plates appear to have sharper contours than before calcination. Other changes, including growth or further aggregation of the plates have not been observed. EDXS and AAS again only detect Si, Zn, and O (EDXS) or Si and Zn (AAS), respectively, and therefore support our XRD structure assignment summarized in Table 1.

During the mineralization process an oily second phase develops on the surface of the reaction solution. NMR reveals that the oil is tributylamine. As the tetrabutylammonium cation eliminates 1-butene at high temperatures [82], the formation of the oily tributylamine phase is not surprising. As amines (although usually primary or secondary amines) can coordinate to transition metals, it is possible that some organic material is incorporated into the zinc silicates formed here. However, both thermal and elemental analysis as well as SSNMR and infrared (IR) spectroscopy show no evidence for incorporation of organic material.

**Figure 7:**
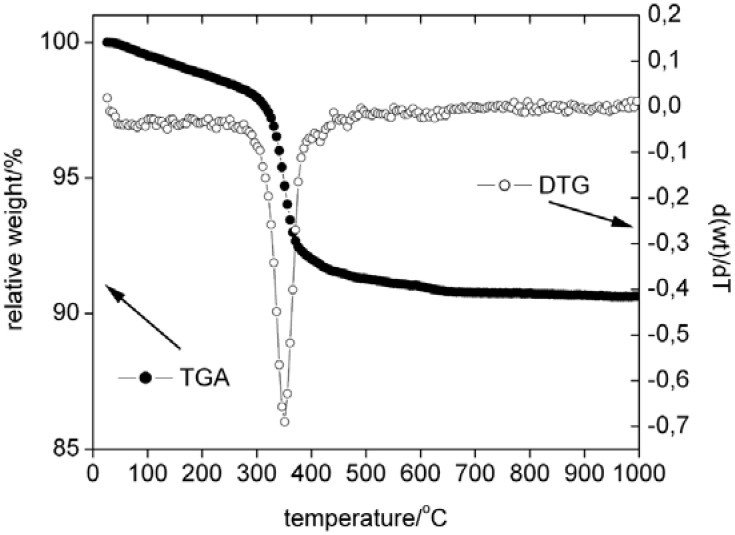
TGA and DTG curves of the as-prepared sample in air. Note that the relative weight scale (TGA) is only from 85 to 101 wt%.

Figure 7 shows thermogravimetric analysis (TGA) and derivative (DTG) data from an as-precipitated sample. TGA curves show a slow but continuous weight loss from room temperature to ca. 350 °C, which we assign to the loss of bound surface water and possibly to the dehydration of γ-zinc hydroxide Zn(OH)_2_ to zincite ZnO and the beginning transformation of hemimorphite to willemite. This is supported by XRD measurements (Figure 1), which find that already calcination at 300 °C transforms the Zn(OH)_2_ into ZnO, while the complete transformation of hemimorphite to willemite requires higher temperatures.

The TGA curves only show one sharp weight loss of 8 to 10 % of the original mass at 350 °C, which is followed by another, very slow weight loss up to 1000 °C. This indicates that a single process is responsible for the weight loss at 350 °C, presumably the transition from hemimorphite to willemite. The subsequent minor and very slow weight loss is assigned to the fact that complete transformation to willemite requires temperatures of 600 °C and more and the dehydration to willemite hence continues up to 1000 °C in the dynamic TGA experiment.

Elemental analysis finds only 0.647 ± 0.006 % of N, 0.283 ± 0.002 % of C, and 0.607 ± 0.006 % of H. The presence of these elements can be assigned to the incorporation of some organic material. These data further confirm that the weight losses observed in TGA are not due to incorporation of organic material into the minerals, but much rather that the above explanation of dehydration and phase transitions applies.

### Crystallization and morphogenesis.

The formation of such complex nano- to microscale materials often follows a more or less intricate multistep pathway. For example, Cölfen and colleagues have recently pointed out that the formation of calcium carbonate particles can either proceed via classical crystallization, that is, the attachment of single ions to an existing crystal surface, or via different non-classical pathways, which lead to meso- or polycrystals [69,70,71,72,73,74,75,76,77,78,83]. In order to elucidate the growth of our complex silicate particles, we have performed time-resolved crystallization experiments.

**Figure 8:**
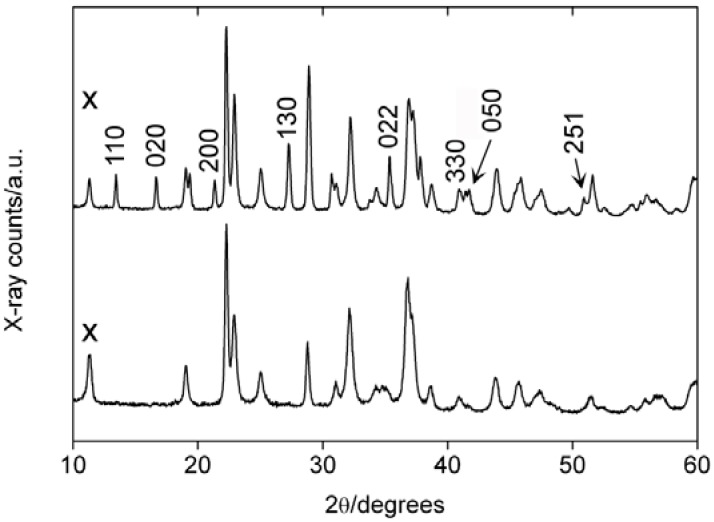
XRD patterns of an as-prepared sample isolated after 20 hours (bottom) and after 30 hours (top). Only the reflections appearing between 20 and 30 hours of reaction time are labeled. (x) denotes the γ-Zn(OH)_2_ 110 reflection, which is only found in the as-precipitated samples.

Figure 8 shows representative XRD patterns from samples isolated after 20 and 30 hours. Both patterns are essentially the patterns of hemimorphite and willemite, but a more detailed analysis shows that some hemimorphite reflections only appear late in the particle formation process. With the exception of 022 and 251, all reflections appearing late are h00, hk0 or 0k0 reflections. This suggests that the particles first grow preferentially along the crystallographic a- and b-axes and only after some time, further growth along the c-axis becomes more pronounced. Such a growth mechanism would also account for the platelike morphology, where the a- and b-axes run along the long and short axes of the hemimorphite nanocrystals. The c-axis would then be parallel to the shortest particle axis, the thickness of the plates. As stated above, we have not been able to obtain clear electron diffraction patterns to confirm this hypothesis, but further experiments in combination with Rietveld refinement are underway.

**Figure 9:**
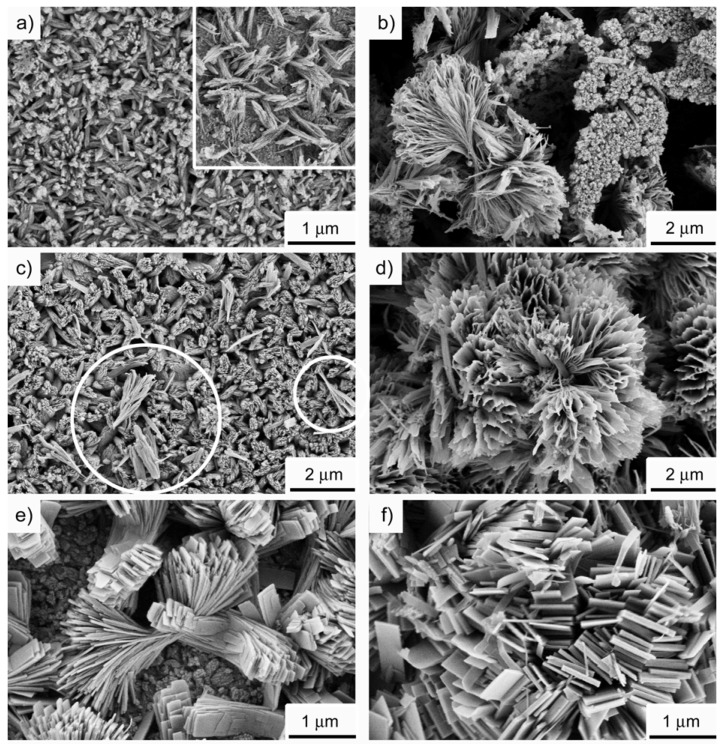
SEM of samples isolated after different reaction times. (a) 10 hours, (b) 16 hours, (c, d) 26 hours, (e) 20 hours, (f) 30 hours. Inset in (a) is a magnified view of the “ellipsoidal” first generation particles, which are made up of threadlike primary building blocks. The circles in (c) highlight a first generation of threads that start to form a wedge (see text for details).

Figure 9 shows SEM images of powders isolated after different reaction times, which support the XRD data, as the first particles have acicular or thread-like shapes and only later the characteristic plate morphology is observed. The images clearly show that the morphology of the particles observed after 30 and more hours of reaction time is preceded by other particle morphologies and forms through various intermediate stages. Initially, small rodlike particles are observed, but soon thereafter, more complex shapes evolve. The morphogenesis is complex and several steps appear to strongly overlap. For example, a fraction of the samples obtained after 20 hours of reaction time already exhibits the morphology, which is also found after 30 and more hours (Figure 9 e). However, some fraction of the material obtained after 26 hours still resembles the sample observed after 10 or 16 hours (Figure 9 c, d). After 30 and more hours of reaction time, the individual plates making up the final, larger particles are essentially identical to plates observed after 20 or 25 hours, only the edges have developed further to yield well-defined platelike crystals.

SEM also reveals that the final particles most likely do not exclusively grow via self-similar crystallization. After short reaction times, we only observe small particles. Later, we also observe particles that appear to have grown from a rodlike primary particle but exhibit a first set of fine threadlike features, which overall have a wedge-like shape. These features become more complex and exhibit 2D and 3D assemblies of threads which overall have a dumbbell shape. However, in parallel to the formation of the assembly and growth of the threads to wedges and finally dumbbells, the individual threads also appear to evolve from a threadlike to a platelike morphology (Figure 9 f).

### Location of particle nucleation and effect of reaction temperature

SEM shows that parts of all samples have a flat bottom. Furthermore, the majority of the sample does not grow from solution, but forms a rather thick white film on the glass wall of the flasks, while the solution remains almost clear. Even after thorough cleaning, the glass flasks remain somewhat turbid. These observations suggest that the silicates form by direct reaction of TBAH, Zn^2+^, and the glass wall. This is further supported by the fact that no additional silicon source was added and by control experiments, which rule out contamination from silicon grease and other third sources.

As the reaction of hydroxide ions with glass is well known, the reaction of TBAH with the glass is not surprising as such. The interesting point, however, is that this approach represents to date the simplest approach for the fabrication of complex zinc silicate nanomaterials with tunable morphologies. As shown above, morphologies can be tuned by simple variation of the reaction time.

**Scheme 1:**
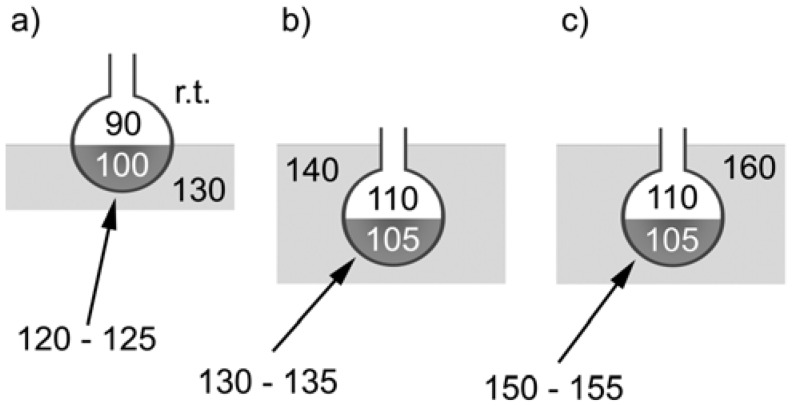
Different reaction conditions that lead to different morphologies of the precipitate. (a) Mineralization of ZnO hollow tubes on the glass wall above the reaction mixture [44]. (b) Formation of the silicate platelets, Figure 3 to Figure 5. (c) Formation of the silicate “sponges”, Figure 10 below. Numbers are temperatures in °C. Arrows denote the temperatures of the glass walls of the flasks.

Alternatively, morphologies can also be tuned by the reaction temperature. All samples discussed so far were grown at a temperature of the glass wall of 130 to 135 °C. This is higher than the temperature of the glass wall in our earlier study (120 to 125 °C), where single phase zincite hollow rods were obtained [44]. Furthermore, in the current study, the glass flasks were immersed into the oil bath in order to also achieve a higher temperature of the glass wall above the liquid phase. This results in a different thermal environment, which dramatically affects the outcome of the reaction. Scheme 1 summarizes the different reaction conditions that lead to the different morphologies observed in the precipitates.

**Figure 10:**
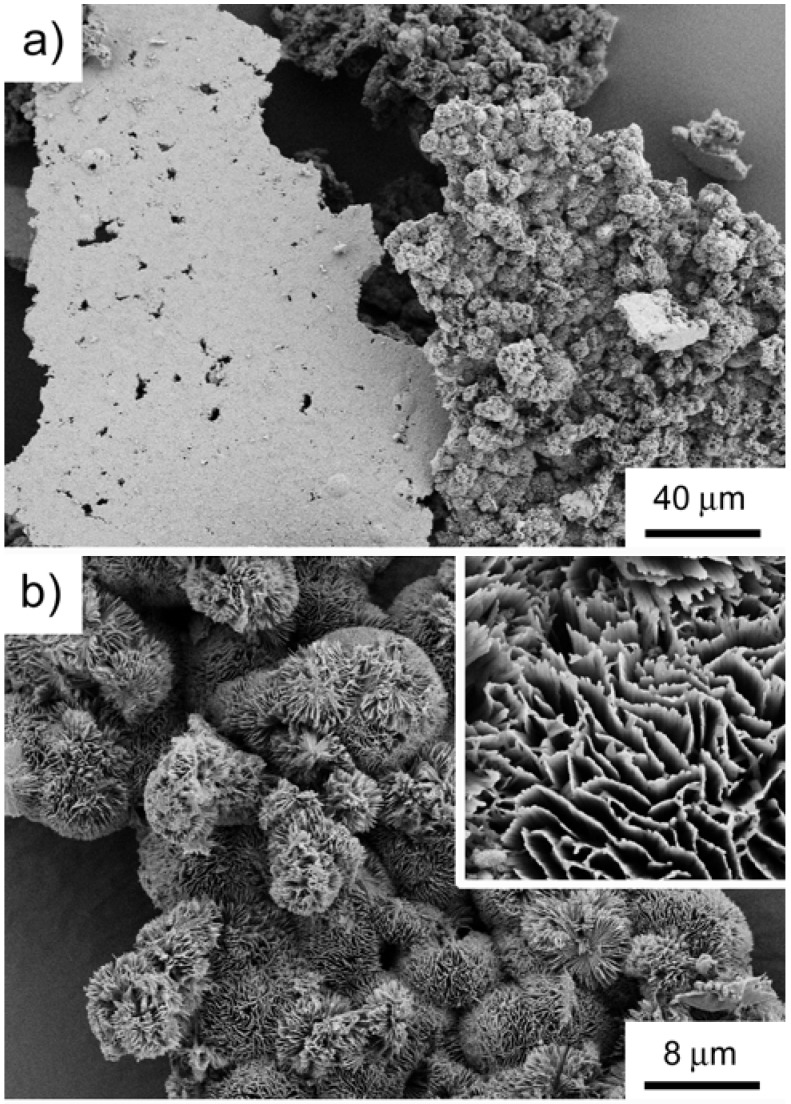
SEM image of a sample obtained at 150 to 155 °C after 30 hours. Panel (a) shows two large pieces of the white film isolated after mineralization. The left part shows the flat bottom and the right part is a piece of the film with the upper side upwards. Panel (b) is a magnified view of the right area in (a). The inset is a magnified view of the lamellae forming the “sponges”.

Figure 10 is an example of a sample grown at 150 to 155 °C (panel c in Scheme 1), which shows that reactions conducted at higher temperatures yield a different morphology of the precipitate. Unlike samples grown at lower temperatures, the samples obtained here do not exhibit the typical plate morphology, but rather have a flower- or sponge-like morphology, where the individual sponges are composed of fine lamellae that are closely connected and form a dense array of “tubular” units.

The observation that the solution is only slightly turbid in all cases is explained by the fact that most of the mineralization takes place as a heterogeneous growth process on the glass wall of the flask. The slight turbidity is most likely due to the formation of γ-Zn(OH)_2_ nanoparticles, which do not form on the glass wall, but in solution. However, as the amount of precipitate recovered from the solution is very small, an unambiguous assignment of its structure is not possible.

### Suggested growth mechanism

The formation of the zinc silicate particles from TBAH is a complex process. Despite this, SEM, SSNMR, and XRD allow for the development of a first growth model. The fact that zinc silicates and not zinc oxide forms, the presence of large particle agglomerates with a flat bottom, the formation of a thick white film on the glass wall, and the fact that the solution is only slightly turbid during the reaction suggests that nucleation and growth of the zinc silicate takes place at the hot glass wall of the reaction flask. SEM shows that initially, small ellipsoidal particles composed of thread-like primary building blocks form. As the reaction proceeds, the particles grow into wedge- and dumbbell-like particles, which are still composed of small threads. These threads then evolve into sheet-like elements, which become the building blocks of 3D flowerlike structures. These finally recrystallize to form the rectangular plates that make up the final particles.

**Scheme 2:**
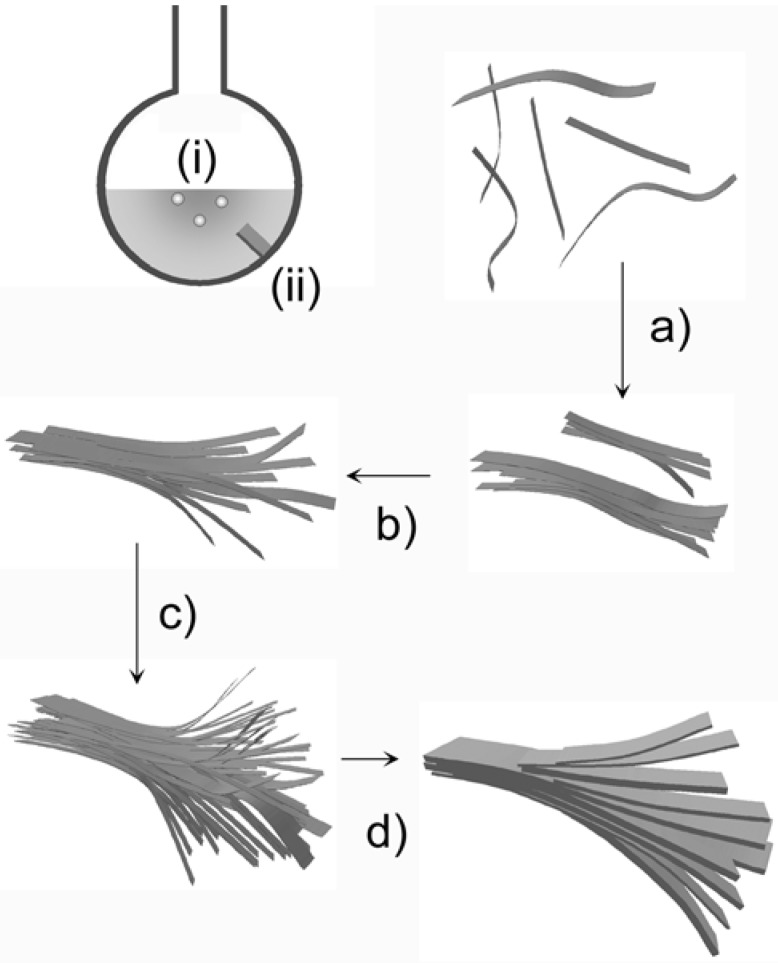
Suggested growth mechanism at ca. 130 °C. (i) Zn(OH)_2_ nucleates in solution and essentially persists as small particles in suspension throughout the reaction. (ii) Hemimorphite nucleates on the glass wall of the flask. (a) Initially, small hemimorphite threads form (Figure 8 a, inset) and relatively quickly aggregate into bundles of threads, which (b) then aggregate further into larger bundles (Figure 8 c, circles). These aggregates transform (c) into the more complex “flowerlike” aggregates (Figure 8 d), which then (d) further transform into the final larger complex structures of nano- to micrometer-sized plates. Drawings are not to scale.

Overall, SEM provides evidence for a combination of self-similar growth processes with self-assembly of smaller particles (ellipsoids into threads into larger 2D and 3D structures). SEM also provides evidence that once the skeleton of the final morphology exists, further particle growth most likely occurs via heterogeneous nucleation on the existing skeleton or via redissolution-reprecipitation of parts of the sample. As SSNMR shows that the dominating species is hemimorphite, it is likely that morphogenesis is largely controlled by hemimorphite and not by willemite growth. Scheme 2 illustrates the proposed growth model.

## 3. Experimental Section

### Mineralization

In a typical experiment, tetrabutylammonium hydroxide (TBAH, N(C_4_H_9_)_4_OH·30H_2_O, Aldrich 86866, m.p. 26 – 28 °C, 5 g) was heated in a 25 mL flask to *ca*. 50 °C until the IL was liquid. Then the flask was cooled to *ca*. 30 °C by immersing in a water bath for a few minutes. Subsequently, Zn(OAc)_2_ dihydrate (Aldrich, 50 mg) was added and the mixture was sonicated until the solution was clear. The flask was immersed in the oil bath such that also the volume of the flask above the reacting liquid was immersed in the oil bath. The samples were refluxed for 30 h. The temperature of the reaction solution was 105 °C and the vapor phase was 105 to 110 °C. This is in contrast to our earlier study [44], where the temperature of the reaction solution was 100 °C and the vapor phase had a temperature of only 90 °C. The glass wall temperature inside the flask was 130 to 135 °C, which is higher than the 120 °C measured in our earlier study [44]. After eight to ten hours, a white precipitate appeared and the solutions remained slightly turbid until the end of the reaction. After ca. twelve hours, a thick white film formed on the glass walls of the flask. After 30 hours, the white solid was recovered via centrifugation, washing with water and ethanol, and drying at 60 °C.

### Characterization

X-ray diffraction was done on a Nonius PDS 120 with CuKα radiation and position sensitive detector and on a Nonius D8 with CuKα radiation. SEM was done on a Philips XL-30 ESEM operated at 10 kV and on a LEO 1550 Gemini operated at 20 kV. Samples were sputtered with Au or Pt (Philips) or Au/Pt (LEO) prior to imaging. EDX spectra were acquired with an EDAX DX-4 system. TEM was done on a Zeiss 912 Omega operated at 120 kV. Thermogravimateric analysis (TGA) was performed on a Netzsch 209 thermobalance. The samples were examined at a heating rate of 5 °C/min in air. Atomic absorption spectroscopy (AAS) was done on a Jobin Yvon ICP-OES JY 38 Plus.

Solid-state NMR experiments were conducted at 9.4 T using a Bruker DSX-400 spectrometer equipped with a ^1^H/X/Y probe using zirconia rotors of 4 mm in diameter. ^1^H MAS NMR spectra were acquired at 400.16 MHz with a ^1^H π/2 pulse length of 2.8 μs at an MAS rate of 15 and 30 kHz and a recycle delay of 15 s. Fast MAS NMR spectra were recorded using a ^1^H/X probe-head using zirconia rotors of 2.5 mm in diameter. The position of the ^1^H resonances is quoted in ppm from external TMS.

^1^H-^29^Si cross polarization (CP) MAS NMR spectra were acquired at 400.16 MHz for ^1^H and 78.5 MHz for ^29^Si at an MAS rate of 10.0 kHz. A ^1^H π/2 pulse length of 3.1 μs, a recycle delay of 10 s, and TPPM decoupling were used during acquisition. The CP contact time was set to 2.0 ms with the Hartmann–Hahn matching condition set using kaolinite. The ^29^Si chemical shifts are quoted in ppm with respect to TMS.

^29^Si single pulse (SP) MAS spectra were acquired at 78.5 MHz at an MAS rate of 10.0 kHz. A ^29^Si π/3 pulse length of 2.3 μs with a recycle delay of 240 s without proton decoupling, were used for acquisition. The ^29^Si chemical shifts are quoted in ppm with respect to TMS.

The ^1^H-^29^Si VCT (variable contact time) CP MAS NMR was conducted using the same conditions as the ^1^H-^29^Si CP MAS with contact times ranging from 0.05 to 15.0 ms. The data was fitted according to the I-S model using equation 1.^84^ The parameter, *T_IS_* is the CP time constant and is a measure of CP magnetization transfer efficiency from spin *I* (^1^H) to spin *S* (^29^Si). No decay of intensity of peaks has been observed and the kinetics curves were analyzed using a simplified equation:

(1) $$I(t)=I_{0}\left(1-\exp(-\frac{t}{T_{CP}})\right)$$

2D ^1^H-^29^Si Heteronuclear correlation (HETCOR)^85^ MAS NMR spectra were acquired at 400.16 MHz for ^1^H and 78.5 MHz for ^29^Si with an MAS rate of 10 kHz. The 2D HETCOR experiments used Frequency-Switched Lee-Goldburg (FSLG) [86] homonuclear decoupling with a ^1^H *rf* field of *ca.* 80.6 kHz in *t*_1_ and ramp-amplitude ^1^H-^29^Si cross-polarization with a contact time of 2 ms. TPPM decoupling [87] was used during acquisition at a decoupling strength of *ca.* 80.6 kHz. The sample volume was restricted to the middle of the rotor to improve the *rf* homogeneity. States-TPPI was employed for phase sensitive detection. The recycle delay was set at 3.0 s. 256 increments were recorded in *t*_1_ to cover the full ^1^H spectral width, with 600 scans acquired in *t*_2_ per increment.

## 4. Conclusions

The current study is an extension of our earlier work, where we showed that the ionic liquid precursor (ILP) TBAH can be exploited for the fabrication of ZnO with different morphologies and optical properties [44,46]. The paper shows that not only ZnO can be grown from the IL TBAH. Also more complex compounds like zinc silicates can be fabricated in a controlled fashion by exploiting the fact that at high temperatures the IL reacts with glass. Although reaction with the glass wall of a reaction flask is not typical for materials chemistry, the paper clearly shows that interesting surface layers can be generated if the conditions are appropriately chosen and well-defined.

With one exception [59], only spherical zinc silicate particles, which tend to aggregate upon calcination, have been reported [58]. The current procedure therefore provides access to other, potentially interesting, hemimorphite and willemite morphologies. They can easily be tuned by variation of the reaction time and temperature. Furthermore, the particles are stable with respect to calcination and do not aggregate further or change their morphology when heated. As a result, our approach is a viable pathway towards non-spherical zinc silicates with complex morphologies with useful properties like high thermal stability and high porosity. As hemimorphite has a strong pyroelectric coefficient, low permittivity and loss, and weak piezoelectricity [81], hemimorphite with the high surface areas accessible with our approach could for example be an appealing sensor material.
